# Supplementary material for: HIV skews the SARS-CoV-2 B cell response towards an extrafollicular maturation pathway
Source: eLife. 2022 Oct 27;11:e79924. doi: 10.7554/eLife.79924 (PMC9643005; doi:10.7554/eLife.79924)
Supplement: Supplementary file 1. [file elife-79924-supp1.docx]

**Supplementary File 1. Flow Cytometry B cell phenotyping antibody panels**

| **Panel** | **Marker** | **Label** | **clone** | **cat no** | **Supplier** |
| --- | --- | --- | --- | --- | --- |
| Core | L/D | APC-Cy7 |  | L10119 | Invitrogen |
|  | CD45 | APC | HI30 | 304012 | BioLegend |
|  | CD3 | Bv711 | OKT3 | 317328 | BioLegend |
|  | CD14 | Bv711 | M5E2 | 301838 | BioLegend |
|  | CD19 | Bv605 | HIB19 | 302244 | BioLegend |
|  | CD27 | Bv510 | O323 | 302836 | BioLegend |
|  | CD38 | PECy7 | HIT2 | 303516 | BioLegend |
|  | IgM | PerCP/Cy5.5 | MHM-88 | 314512 | BioLegend |
|  | IgD | AF700 | IA6-2 | 348230 | BioLegend |
| Homing | CD27 | Bv510 | O323 | 302836 | BioLegend |
|  | CCR6 (CD196) | Bv421 | GO34E3 | 353439 | BioLegend |
|  | CXCR5 | AF488 (FITC) | RF8B2 | 558112 | BD Pharmingen |
|  | CXCR4 (CD184) | Bv785™ | 12G5 | 306530 | BioLegend |
|  | CD62L | PE-Cy5 | DREG-56 | 555545 | BD Pharmingen |
|  | CXCR3 (CD183) | PE-CF594 | IC6/CXCR3 | 562451 | BD Horizon |
|  | CD69 | BUV395 | FN50 | 564364 | BD Horizon |
|  | CCR7 | PE | 150503 | FAB197P | R&D Biosystems |
| Maturation | CD27 | Bv510 | O323 | 302836 | BioLegend |
|  | CD138 (Syndecan-1) | Bv785™ | MI15 | 356538 | BioLegend |
|  | CXCR5 | AF488 (FITC) | RF8B2 | 558112 | BD Pharmingen |
|  | CD11c | PE | S-HCL-3 | 371504 | BioLegend |
|  | CD95 (Fas) | Bv650™ | DX2 | 305642 | BioLegend |
|  | CD20 | PE/Dazzle™ 594 | 2H7 | 302348 | BioLegend |
|  | CD69 | BUV395 | FN50 | 564364 | BD Horizon |
|  | CD10 | PE-Cy5 | HI10a (RUO) | 555376 | BD Pharmingen |
|  | CD21 | Bv421 | B-ly4 | 562966 | BD Horizon |
|  | CD40 | BUV496 | 5C3 | 741159 | BD OptiBuild |
| Regulatory | CD27 | PE-Cy5 | 1A4CD27 | 6607107 | Beckman Coulter |
|  | CD40 | BUV496 | 5C3 | 741159 | BD OptiBuild |
|  | PD-L1 (CD274) | PE | 29E.2A3 | 329706 | BioLegend |
|  | CD24 | FITC | ML5 | 311104 | BioLegend |
|  | CD178 (Fas-L) | Bv421™ | NOK-1 | 306412 | BioLegend |
|  | CD1d | Bv510™ | 51.1 | 350314 | BioLegend |
|  | CD5 | PE/Dazzle™ 594 | L17F12 | 364012 | BioLegend |
|  | CD86 | Bv650™ | IT2.2 | 305428 | BioLegend |
