# Supplementary material for: HIV skews the SARS-CoV-2 B cell response towards an extrafollicular maturation pathway
Source: eLife. 2022 Oct 27;11:e79924. doi: 10.7554/eLife.79924 (PMC9643005; doi:10.7554/eLife.79924)
Supplement: Supplementary file 2. [file elife-79924-supp2.docx]

**Supplementary File 2. Flow Cytometry B cell BAIT antibody panel**

| **Marker** | **Label** | **clone** | **cat no** | **Supplier** |
| --- | --- | --- | --- | --- |
| L/D | APC-Cy7 |  | L10119 | Invitrogen |
| CD45 | Hv500 | HI30 | 560777 | BD Horizon |
| CD3 | Bv711 | OKT3 | 317328 | BioLegend |
| CD14 | Bv711 | M5E2 | 301838 | BioLegend |
| CD19 | Bv605 | HIB19 | 302244 | BioLegend |
| CD27 | PE-Cy5 | 1A4CD27 | 6607107 | Beckman Coulter |
| CD38 | PECy7 | HIT2 | 303516 | BioLegend |
| IgM | PerCP/Cy5.5 | MHM-88 | 314512 | BioLegend |
| IgD | AF700 | IA6-2 | 348230 | BioLegend |
| CXCR3 | PE-CF594 | IC6/CXCR3 | 562451 | BD Horizon |
| CD21 | Bv421 | B-ly4 | 562966 | BD Horizon |
| BAIT | SA-APC |  | 405207 | BioLegend |
| BIAT | SA-PE |  | 405204 | BioLegend |
